# Machine learning-enhanced echocardiography for screening coronary artery disease

**DOI:** 10.1186/s12938-023-01106-x

**Published:** 2023-05-11

**Authors:** Ying Guo, Chenxi Xia, You Zhong, Yiliang Wei, Huolan Zhu, Jianqiang Ma, Guang Li, Xuyang Meng, Chenguang Yang, Xiang Wang, Fang Wang

**Affiliations:** 1 Department of Cardiology, Beijing Hospital, National Center of Gerontology; Institute of Geriatric Medicine, Chinese Academy of Medical Sciences, Beijing, 100730 People’s Republic of China; 2 Jiangsu Key Laboratory of Phylogenomics and Comparative Genomics, School of Life Sciences, Jiangsu Normal University, Xuzhou, 221116 Jiangsu People’s Republic of China; 3 Department of Immunology, Biochemistry and Molecular Biology, 2011 Collaborative Innovation Center of Tianjin for Medical Epigenetics, Tianjin Key Laboratory of Medical Epigenetics, Tianjin Medical University, Tianjin, 300070 People’s Republic of China; 4 Department of Gerontology, Shaanxi Provincial People’s Hospital, Shaanxi Provincial Clinical Research Center for Geriatric Medicine, No. 256 Youyi West Road, Xi’an, China; 5 Keya Medical Technology Co., Ltd, Beijing, People’s Republic of China

**Keywords:** Coronary artery disease, Myocardial work, Machine learning, Left atrial strain

## Abstract

**Background:**

Since myocardial work (MW) and left atrial strain are valuable for screening coronary artery disease (CAD), this study aimed to develop a novel CAD screening approach based on machine learning-enhanced echocardiography.

**Methods:**

This prospective study used data from patients undergoing coronary angiography, in which the novel echocardiography features were extracted by a machine learning algorithm. A total of 818 patients were enrolled and randomly divided into training (80%) and testing (20%) groups. An additional 115 patients were also enrolled in the validation group.

**Results:**

The superior diagnosis model of CAD was optimized using 59 echocardiographic features in a gradient-boosting classifier. This model showed that the value of the receiver operating characteristic area under the curve (AUC) was 0.852 in the test group and 0.834 in the validation group, with high sensitivity (0.952) and low specificity (0.691), suggesting that this model is very sensitive for detecting CAD, but its low specificity may increase the high false-positive rate. We also determined that the false-positive cases were more susceptible to suffering cardiac events than the true-negative cases.

**Conclusions:**

Machine learning-enhanced echocardiography can improve CAD detection based on the MW and left atrial strain features. Our developed model is valuable for estimating the pre-test probability of CAD and screening CAD patients in clinical practice.

*Trial registration*: Registered as NCT03905200 at ClinicalTrials.gov. Registered on 5 April 2019.

## Introduction

Early diagnosis of coronary artery disease (CAD), a leading cause of death worldwide [1], is an effective strategy to decrease the mortality of CAD patients and improve their prognosis. In recent years, several novel non-invasive imaging techniques have been applied in diagnosing CAD, including electrocardiogram, CT and cardiac MRI [2–6], but echocardiography is still a first-line diagnostic tool for CAD [7] owing to its feasibility and reliability. Nevertheless, the gold standard for CAD diagnosis in the clinic is coronary angiography [7], but this common imaging modality is complicated and costly and has side effects. Therefore, developing high-sensitivity models that can be used for the clinical screening of CAD patients, especially those with the integration of artificial intelligence (AI) and noninvasive imaging techniques, is urgently needed.

The myocardial work (MW) is one of the newly developed noninvasive techniques for CAD diagnosis, which has an assessment function of deformation and afterload that provides incremental value to the evaluation of cardiac function [8–10]. It also has better sensitivity and accuracy in detecting cases with single- or multivessel CAD, as compared to left ventricular ejection fraction (LVEF) and global longitudinal strain (GLS) [11], in particular the close correlation of MW and invasive coronary angiography in the measurement of coronary stenosis severity [12]. MW might be a new and sensitive tool for screening patients with CAD. Previous studies revealed that left atrial (LA) dysfunction is an early event of CAD, based on the observation that left ventricular (LV) diastolic dysfunction may occur before LV systolic dysfunction in case of ischemia [13]. Moreover, the fibrotic processes in the LA could cause LA functional alterations in CAD patients. LA dysfunction appears in the early stage of CAD [14, 15]. Therefore, echocardiography has a better diagnostic value for detecting LA dysfunction than conventional methods [16].

Although the novel noninvasive tools hold new promise for screening CAD patients, they are not perfect, because they lack the computational models that can combine these new methods to improve diagnostic performance in their clinical application. AI technology has been widely used to diagnose, treat, and manage different diseases [17–23]. Therefore, it is necessary to develop novel diagnostic models for non-invasive tools using AI technology. Machine learning (ML) algorithm may solve the problem, demonstrating its influence on diagnostic decision-making [24–31]. Nevertheless, more investigations in this field are still needed to improve the combination of ML algorithms with new clinical approaches. Previous AI studies did not focus on noninvasive and novel ultrasound tools, most of which only included conventional ultrasound parameters. Moreover, much data related to MW have not been analyzed in previous studies. To this end, this study aimed to establish an effective ML model based on novel echocardiography features, which may enable the procedure of CAD diagnosis to be more sensitive, accurate, and simplified, because we believe that the MI-based models can extract the echocardiography features more effectively for improving the accuracy of echocardiography, reducing the input of experts, saving both cost and time, and ultimately providing high-quality services for patients. Furthermore, it would be appealing to evaluate the diagnostic value of CAD using this machine-learning model.

## Methods

### Patients

This prospective clinical trial (NCT03905200, registered on 5 April 2019) included 958 cases diagnosed as clinically suspected CAD by coronary angiography in Beijing Hospital, Beijing, China. The patients with CAD were diagnosed by coronary angiography, which showed  ≥ 50% stenosis in one or more coronary arteries [32]. The inclusion criteria used for this study included (1) patients with typical myocardial ischemia-related symptoms (such as shortness of breath, chest tightness, chest pain, and palpitation) or positive results of examinations; (2) 18 years and above; (3) with sinus rhythm. The exclusion criteria were patients with (1) obstruction or pressure gradient between the aorta and LV; (2) severe valvular heart disease or arrhythmia; (3) other extremely severe organ illnesses; (4) echocardiographic images with poor quality for speckle tracking. Finally, 818 patients were recruited in this study, approved by the Beijing Hospital ethics committee (reference number: 2020BJYYEC-021-02), and followed the Declaration of Helsinki.

### Echocardiography

The instruments for echocardiography included Vivid E9 and Vivid E95 ultrasound systems (GE Vingmed Ultrasound, Horten, Norway). The baseline echocardiography for the admitted patients was implemented prior to their coronary angiography. The original data of echocardiography images were stored in DICOM format. EchoPac software (EchoPAC 204, GE Vingmed Ultrasound) was used to analyze conventional, MW, LA indices offline. The performance guidelines of echocardiography were the American Society of Echocardiography guidelines [33, 34]. The cardiac images of the echocardiogram, having the best visualization of the myocardium, were used for further analysis. Biplane Simpson’s method was used for calculating LVEF. Echocardiographic GLS is an important parameter indicating the left ventricle deformation in the longitudinal direction [35]. Echocardiography is helpful in evaluating LV function, especially using the LV MW parameters, including the global myocardial work index (GWI), global constructive work (GCW), global wasted work (GWW), and global work efficiency (GWE).

MW indices were determined by the EchoPAC software, which had a pressure–strain loop area module made from non-invasively estimated LV pressure curves and LV strain. Peak systolic LV pressure was assumed to be equal to the peak brachial cuff systolic blood pressure that was measured simultaneously at the echocardiography examination, which has been reported previously [36–40]. Moreover, the GWI, which can be calculated by the EchoPAC software, represents MW inside the pressure–strain loop area, a novel parameter to assess LV performance. The EchoPAC software can also calculate the additional parameters as follows: the GCW, an MW for shortening during ventricular systole and lengthening during isovolumic relaxation; the GWW, an MW for lengthening during ventricular systole and shortening during isovolumic relaxation; the GWE, the percentage of myocardial constructive work in total MW [GCW/(GCW + GWW)]. Other MW parameters can also be obtained using EchoPAC, such as the global positive work (GPW), the global negative work (GNW), the global systolic constructive work (GSCW), and the global systolic wasted work (GSWW). Other parameters were calculated using MW software based on the standardized myocardial segmentation previously published [33, 41, 42], including 18-segment values of GWI, GWE, constructive work (ConsW), wasted work (WastedW), positive work (PositiveW), negative work (NegativeW), systolic constructive work (SysConsW), systolic wasted work (SysWastedW), and peak systolic strain (PSS).

In addition, the measurement of LA strain was performed using the instrument of AFI LA (EchoPAC 204, GE Vingmed Ultrasound), in which zero strain was defined by the automatic R-wave trigger on the electrocardiogram. EchoPac 204 is equipped with a software package for evaluating LA strain based on the strain values in three phases: reservoir strain in systole (LASr), conduit strain in early diastole (LASct), and contraction strain in late diastole (LAScd).

### Features and data process

**Fig. 1:**
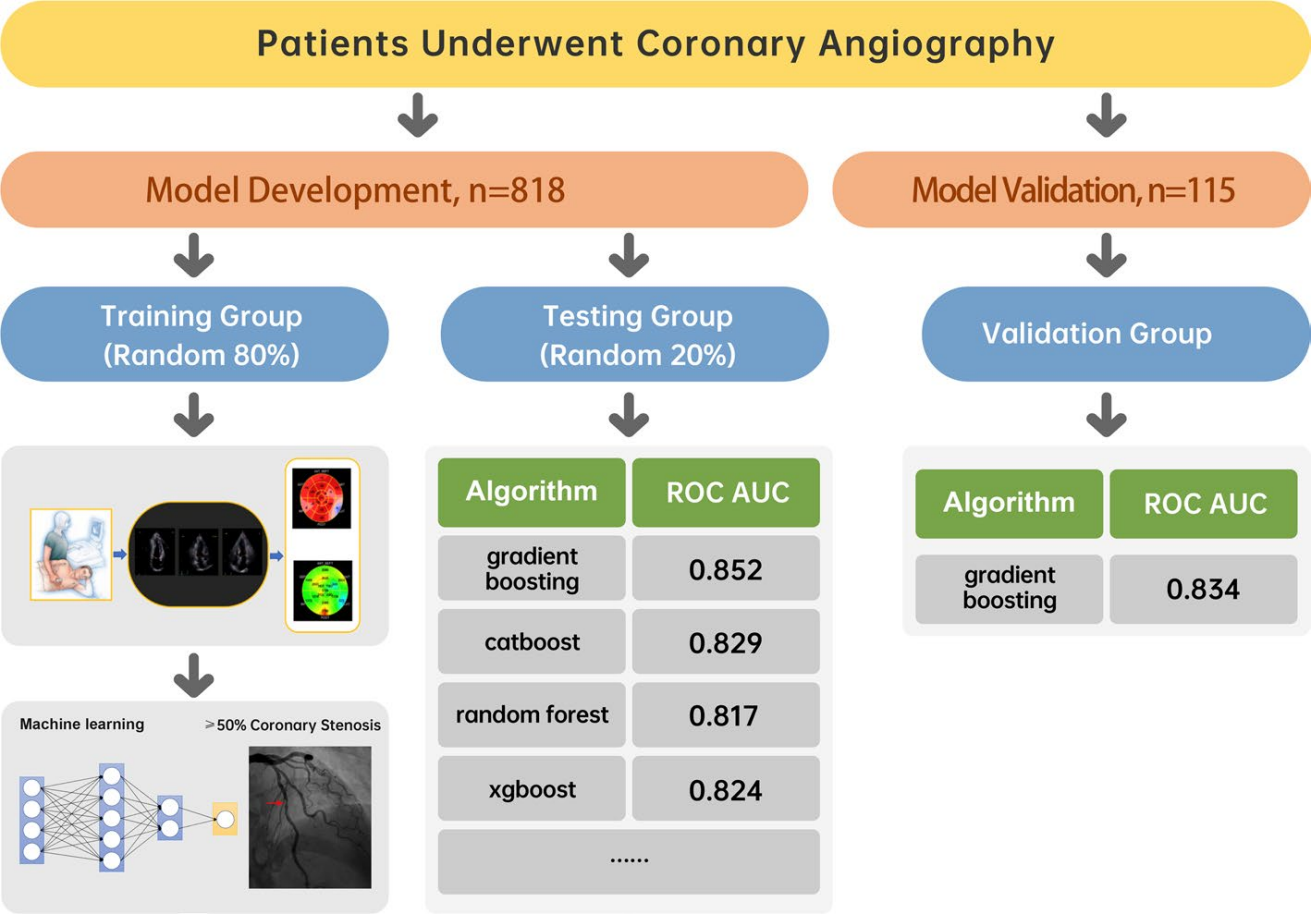
Study flowchart. All patients who underwent coronary angiography were divided into a data set of 818 patients for model training and classifier testing and a validation data set of 115 patients for validating the efficiency of the predictive model. *AUC* Area under the curve, *ROC* Receiver operator characteristics

A flow diagram of this study is illustrated in Fig. 1. A total of 818 subjects received an echocardiography examination 1 day before their angiography. Among 818 patients, only 497 were diagnosed with CAD by coronary angiography (with  ≥ 50% luminal stenosis in one or more major epicardial coronary arteries).

To explore the echocardiographic features for the diagnosis of CAD, the assessment of LV function by MW technology was performed in this study, including the MW method and standard 18-segment model [33, 42, 43]. The MW parameters of the myocardium in 18 segments can be individually calculated. The region of interest thickness could be determined by the LA shape and the thickness of the LA myocardium, thereby avoiding the strong signals of the pericardial tissue. LA strain was measured as GLS of the entire wall, but the segmental LA strain was not considered [44]. As shown in Table 1, 242 features were extracted for predicting the risk of CAD, including MW and LA strain-related features, clinic features, and conventional echocardiographic data. These features included conventional and novel indices. After removing the cases which contained more than 10% of missing data in the process of extracting features, 793 patients and 235 features were reserved for establishing the ML model.

**Table 1:** 242 features chosen for predicting CAD*

MW and LA strain-related features (202)							
*MW features (170)*							
GWE	GWI	GCW	GWW	GPW	GNW	GSCW	GSWW
MW (18 segments)	GWE (18 segments)	ConsW (18 segments)	WastedW (18 segments)	PositiveW (18 segments)	NegativeW (18 segments)	SysConsW (18 segments)	SysWastedW (18 segments)
PSS (18 segments)							
*MW-related features (LV strain features 23)*							
PSD	Peak systolic longitudinal strain (APLAX/A4C/A2C)	GLS	GLS (18 segments)				
*LA strain features (9)*							
LASr (A4C/A2C/avg)	LAScd (A4C/A2C/avg)	LASct (A4C/A2C/avg)					

Clinic features (21)							
Age	Hypertension	Hyperlipemia	Family history	Uric acid	Creatinine	Homocysteine	BMI
Gender	DM	Smoking	Heart rate	LDL-C	FPG	HbA1c	Electrocardiogram
Systolic BP	Diastolic BP	Height	Weight	Body surface area			

Conventional echocardiographic data (19)							
Regional wall motion abnormalities	Aortic sinus diameter	Ascending aorta diameter	Left atrial diameter	Inter-ventricular septum end-diastolic thickness	LV end-diastolic diameter	LV posterior wall end-diastolic thickness	LV end-diastolic volume
LVEF	Right ventricular end-diastolic diameter	Mitral A wave	Mitral E wave	Mitral E/e’ septal average ratio	Tricuspid A wave	Tricuspid E wave	Peak aortic valve velocity
s’ Septal TDI	e’ Septal TDI	a’ Septal TDI					

*BMI* body mass index, *BP* blood pressure, *CAD* coronary artery disease, *ConsW* constructive work, *DM* diabetes mellitus, *FPG* fasting plasma glucose, *GCW* global constructive work, *GLS* global longitudinal strain, *GNW* global negative work, *GPW* global positive work, *GSCW* global systolic constructive work, *GSWW* global systolic wasted work, *GWE* global work efficiency, *GWI* global work index, *GWW* global wasted work, *LA* left atrial, *LAScd* left atrial longitudinal strain during conduit phase, *LASct* left atrial longitudinal strain during contraction phase, *LASr* left atrial longitudinal strain during reservoir phase, *LDL_C* low-density lipoprotein cholesterol, *LVEF* left ventricular ejection fraction, *MW* myocardial work, *NegativeW* negative work, *PositiveW* positive work, *PSD* peak strain dispersion, *PSS* peak systolic strain, *SysConsW* systolic constructive work, *SysWastedW* systolic wasted work, *TDI* tissue doppler imaging, *WastedW* wasted work

^*^7 variables with missing data > 10%, left 235 variables for training model

### ML classifiers

Recently, ML-based research methods have been widely used in clinical diagnostic studies [45]. This study focused on a ML model which can be used for CAD screening with echocardiography features. In this study, different kinds of ML classifiers were applied, such as logistic regression (logistic regression, softmax, and dummy), naïve bayes, k-neighbors, linear classifier (ridge and support vector machine), quadratic discriminant analysis, decision tree bagging (random forest and extra trees), and gradient boosting (catBoost, AdaBoost, light gradient boosting, gradient boosting, and Xgboost) classifiers. Moreover, blend and stack models were established and tested with the five best classifiers. The gradient boosting algorithms demonstrated high efficiency in CAD predictive learning using echocardiography features. The gradient boosting method is a system of joint weak learners (tree- or linear-based learners) that can minimize the loss function in each step of the iterative model. Usually, a superior classifier has been identified to build a training model.

### Model training

The tenfold cross-validation test was also applied for the model training. The CAD data set was randomly divided into two parts (80% training data set and 20% testing data set). A model for CAD prediction was built by extracting the imaging and clinical features with ML methods.

#### Feature selection

The imaging and clinical features were reduced and selected using different strategies to overcome the problem of model overfitting. The importance of variables was evaluated in each classifier. For example, the correlation coefficients (*r* value) between CAD and each feature were calculated. To obtain the best combinations of independent features, 235 candidate variables were screened by the collinearity diagnostics method using the software SPSS v26. The mean decrease accuracy (MDA) of each variable was calculated using the R package “random forest (v4.6-14)”. The rank of the variables was determined by the descending order of the MDA values. The important features derived from different classifiers were gradually combined to promote the model's accuracy. The relationship between decreasing classification accuracy and the increasing number of features was evaluated by a tenfold cross-validation test.

#### Training data set optimization

**Fig. 2:**
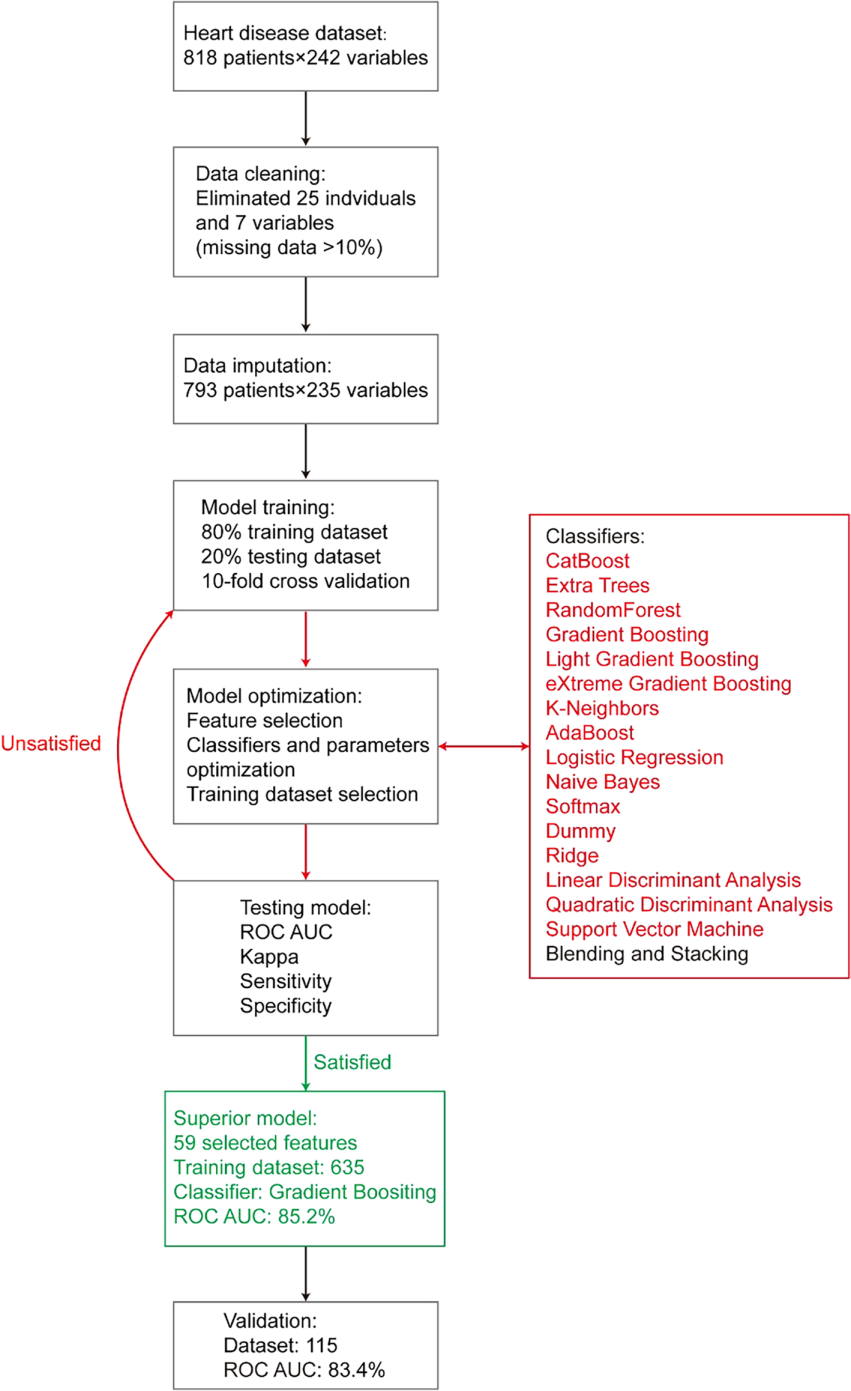
Framework of the proposed model for diagnosing CAD. A stepwise workflow to optimize the predictive learning model by screening and recomposing features, selecting superior classifiers, and identifying the convergent training data set. *AUC* Area under the curve, *ROC* Receiver operator characteristics

Since the randomly divided training data sets also influence the classifier accuracy, we selected a group of high-performance training data sets after hundreds of cross-validation tests. The intersection of various training data sets with an accuracy of around 80 ~ 85% was selected as a standard to build the final training data set for CAD detection. The framework of the proposed model for diagnosing CAD is illustrated in Fig. 2.

#### Model validation

Additional 115 patients recruited from September 2021 to July 2022 were enrolled as the validation data set (out from the cross-validation test), which was used for selecting classifiers and validating the model performance.

### Prognostic follow-up

The follow-up information was obtained through clinical visits or telephone calls by an investigator blind to clinical factors and coronary angiography data. All-cause mortality and cardiovascular hospitalization composited the study endpoint. The death documentation was obtained from hospital medical records and phone conversations with family members. Furthermore, the cause of death was adjudicated by a review of medical records. The follow-up data were obtained up to June 2022. The overall completeness of follow-up was 74.9%. The mean follow-up time was 2.6 years (0.8–3.5 years).

### Statistical analysis

The data of the continuous variables with normal distribution were expressed as mean ± standard deviation, and those without normal distribution were expressed as median (interquartile range). Chi-square or Fisher’s exact tests was used for comparing the difference between categorical variables, while the comparison of continuous variables was performed using the *t* test or Mann–Whitney *U* test. The diagnostic performance of all measures was evaluated based on the exact 95% confidence intervals. Receiver operating characteristic (ROC) analysis was used to evaluate the diagnostic accuracy of parameters. The intraclass correlation coefficients were used for assessing the intra- and inter-observer variabilities of the MW and LA strain parameters. The test results with *P* < 0.05 were considered statistically significant. SPSS 26.0 software was used for the statistical analysis of all data in this study.

## Results

### Clinical and echocardiographic information of CAD patients

A total of 818 patients (mean age, 64.1 ± 9.7 years; 515 men) were enrolled in this study. Among 818 patients, 497 (60.7%) were diagnosed with CAD by coronary angiography. As shown in Table 2, according to the invasive coronary artery angiography, two groups of patients had different echocardiographic characteristics (< 50% or ≥ 50% stenosis of the coronary artery). There were significant differences in the echocardiographic features between the two groups (*P* < 0.05), including GLS, peak strain dispersion (PSD), GWE, GWI, GCW, GPW, GSCW, GSWW, LASr (Avg), and LAScd (Avg), as well as in the clinical features: gender, diabetes, smoking, systolic BP, uric acid, creatinine, electrocardiogram (suspected myocardial ischemia), and LVEF. For MW and LA strain-related features, GWI, GCW, GWE, and LASr (Avg) were significantly higher in non-CAD patients when compared to the CAD patients (*P* < 0.001).

**Table 2:** Summary of clinical and echocardiographic characteristics of the subjects

Characteristic	Total	Coronary Artery Disease (CAD)		*P* value
		Negative (*n* = 321)	Positive (*n* = 497)	
Age, year	64.1 ± 9.7	64.2 ± 9.3	64.1 ± 9.9	0.877
Female, *n* (%)	303 (37.0)	167 (52.0)	136 (27.4)	< 0.001***
Family history of CAD, *n* (%)	246 (30.1)	90 (28.0)	156 (31.4)	0.346
DM, *n* (%)	312 (38.1)	93 (29.0)	219 (44.1)	< 0.001***
Smoke, *n* (%)	381 (46.6)	111 (34.6)	270 (54.3)	< 0.001***
Hyperlipoidemia, *n* (%)	587 (71.8)	223 (69.5)	364 (73.2)	0.276
Hypertension, *n* (%)	547 (66.9)	206 (64.2)	341 (68.6)	0.215
BMI, kg/m^2^	25.9 ± 4.8	25.7 ± 3.6	26.1 ± 5.4	0.274
Systolic BP, mmHg	131.6 ± 15.6	134.2 ± 16.3	129.9 ± 14.9	< 0.001***
Diastolic BP, mmHg	76.7 ± 10.6	77.9 ± 10.8	75.9 ± 10.0	0.505
Electrocardiogram (Suspected myocardial ischemia), n (%)	158 (19.3)	45 (14.0)	113 (22.7)	0.003**
Creatinine, μmol/L	71.7 ± 25.5	67.2 ± 15.9	74.5 ± 29.8	< 0.001***
FPG, mmol/L	6.1 ± 2.7	5.9 ± 3.6	6.2 ± 2.0	0.181
Uric acid, μmol/L	346.7 ± 85.9	335.1 ± 84.5	354.3 ± 86.1	0.002**
LDL-C, mmol/L	2.3 ± 0.9	2.3 ± 0.7	2.3 ± 1.0	0.831
HbA1c, %	6.5 ± 1.0	6.2 ± 0.8	6.6 ± 1.1	< 0.001***
Homocysteine, μmol/L	12.5 ± 6.4	12.2 ± 5.5	12.7 ± 6.9	0.838
LVEF, %	62.6 ± 6.4	64.0 ± 3.8	61.8 ± 7.5	< 0.001***
Left atrial diameter, mm	35.1 ± 4.6	35.0 ± 4.6	35.2 ± 4.5	0.512
Mitral E/e’ ratio	12.1 ± 4.1	12.0 ± 3.7	12.2 ± 4.3	0.652
LV end-diastolic diameter, mm	46.1 ± 4.5	45.7 ± 4.2	46.4 ± 4.7	0.126
Regional wall motion abnormalities, *n* (%)	87 (10.6)	16 (5.0)	71 (14.3)	< 0.001***
GLS, %	−16.6 ± 3.1	−17.9 ± 2.7	−15.9 ± 3.1	< 0.001***
PSD, ms	69.5 ± 33.9	64.5 ± 28.3	72.8 ± 36.8	< 0.001***
GWI, mmHg%	1798.6 ± 419.2	1995.2 ± 393.7	1671.6 ± 385.1	< 0.001***
GCW, mmHg%	2020.2 ± 432.4	2220.8 ± 407.2	1890.5 ± 397.5	< 0.001***
GWW, mmHg%	131.6 ± 101.4	124.7 ± 88.1	136.1 ± 108.9	0.099
GWE, %	0.9 ± 0.1	0.9 ± 0.0	0.9 ± 0.1	< 0.001***
GPW, mmHg%	1981.7 ± 412.9	2178.8 ± 391.4	1854.4 ± 374.9	< 0.001***
GNW, mmHg%	170.1 ± 106.8	166.7 ± 97.3	172.2 ± 112.5	0.459
GSCW, mmHg%	1940.3 ± 420.0	2139.3 ± 394.0	1811.8 ± 385.0	< 0.001***
GSWW, mmHg%	90.3 ± 90.3	85.2 ± 78.3	93.5 ± 97.3	0.181
LASr (Avg), %	28.9 ± 9.3	30.6 ± 9.0	27.8 ± 9.3	< 0.001***
LAScd (Avg), %	−13.8 ± 6.4	−15.0 ± 6.4	−13.0 ± 6.4	< 0.001***
LASct (Avg), %	−15.1 ± 5.9	−15.4 ± 5.5	−14.9 ± 6.2	0.188

*BMI* body mass index, *BP* blood pressure, *CAD* coronary artery disease, *DM* diabetes mellitus, *FPG* fasting plasma glucose, *GCW* global constructive work, *GLS* global longitudinal strain, *GNW* global negative work, *GPW* global positive work, *GSCW* global systolic constructive work, *GSWW* global systolic wasted work, *GWE* global work efficiency, *GWI* global work index, *GWW* global wasted work, *LA* left atrial, *LAScd* left atrial longitudinal strain during conduit phase, *LASct* left atrial longitudinal strain during contraction phase, *LASr* left atrial longitudinal strain during reservoir phase, *LDL_C* low-density lipoprotein cholesterol, *LVEF* left ventricular ejection fraction, *MW* myocardial work, *PSD* peak strain dispersion

^**^P < 0.01, ^***^P < 0.001, compared to negative subjects. Data are expressed as mean ± SD for continuous data or number (percent) for categorical data

The correlation among MW, LA strain parameters, and other numerical features was evaluated. Results showed moderate correlations of GWI, GCW, GPW, and GSCW with systolic BP, LVEF, and PSD (r = 0.379 ~ 0.476, 0.432 ~ 0.444, and −0.259 ~ −0.358, all *P* < 0.01). GWE was also correlated with LVEF and PSD (r = 0.369 and −0.612, both *P* < 0.01). This study also found weak correlations between MW parameters with most serum biochemical indicators and other conventional echocardiographic indices (*P* < 0.05). The LA strain parameters analysis showed mild correlations of LASr with age, LA diameter, and LVEF (r = −0.302, −0.231, and 0.231, *P* < 0.01).

### Building CAD risk prediction model

First, all 235 variables were used for training the model with different classifiers, but the training performance was not satisfied, because the ROC area under the curve (AUC) of 18 classifiers was under 75%, ranging from 49.5% (with quadratic discriminant classifier) to 75.0% (with catBoost classifier). To overcome the model overfitting and promote the diagnostic performance, a selection of features and classifiers was performed, in which, according to correlation coefficients, 33 top-related variables associated with CAD (|*r*|> 0.3) were selected. Meanwhile, a panel of 14 relatively independent variables was left after collinearity diagnostics. Moreover, a panel of 27 top-high MDA variables with the lowest cross-validation error rate was ascertained, and a panel of 39 most important variables was identified across all the tested classifiers. Therefore, the final pool of the selected features included 106 variables, all used to establish the prediction model.

**Fig. 3:**
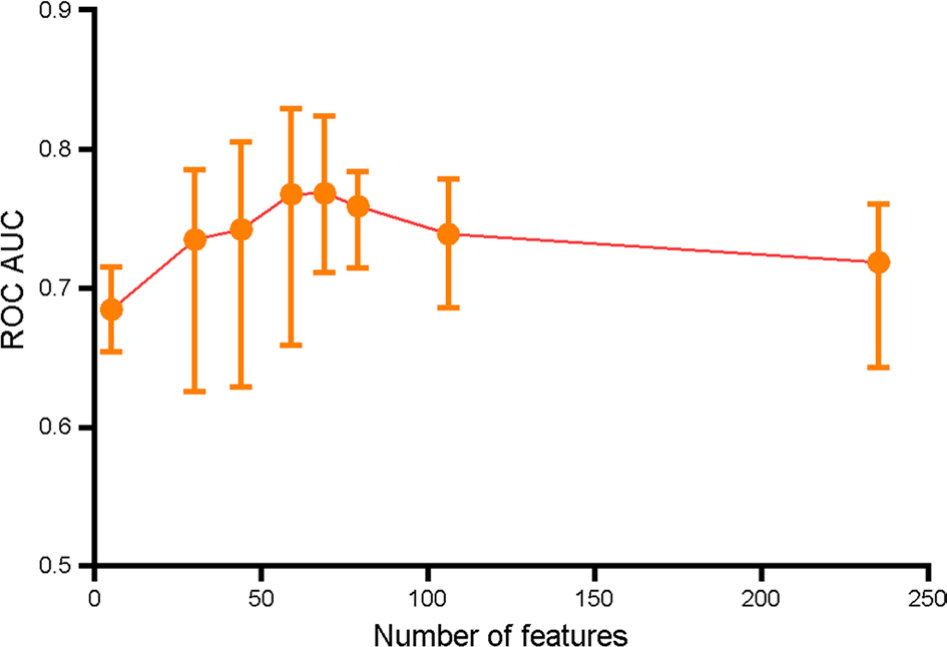
Changes in the curve of ROC AUC with different feature combinations, including the ten best classifiers for each feature combination. Different feature panels screened by MDA (27 features), *r* coefficient (33 features), collinearity diagnostics (14 features), and the importance across all the tested classifiers (39 features). Stepwise combinations of 5 (overlapped features: GWI, G peak SL Full (Avg), Systolic BP., Diastolic BP, LASr R-Wave (Avg)), 30, 44, 59, 69, 79, 106, and 235 features were tested by classifiers

**Fig. 4:**
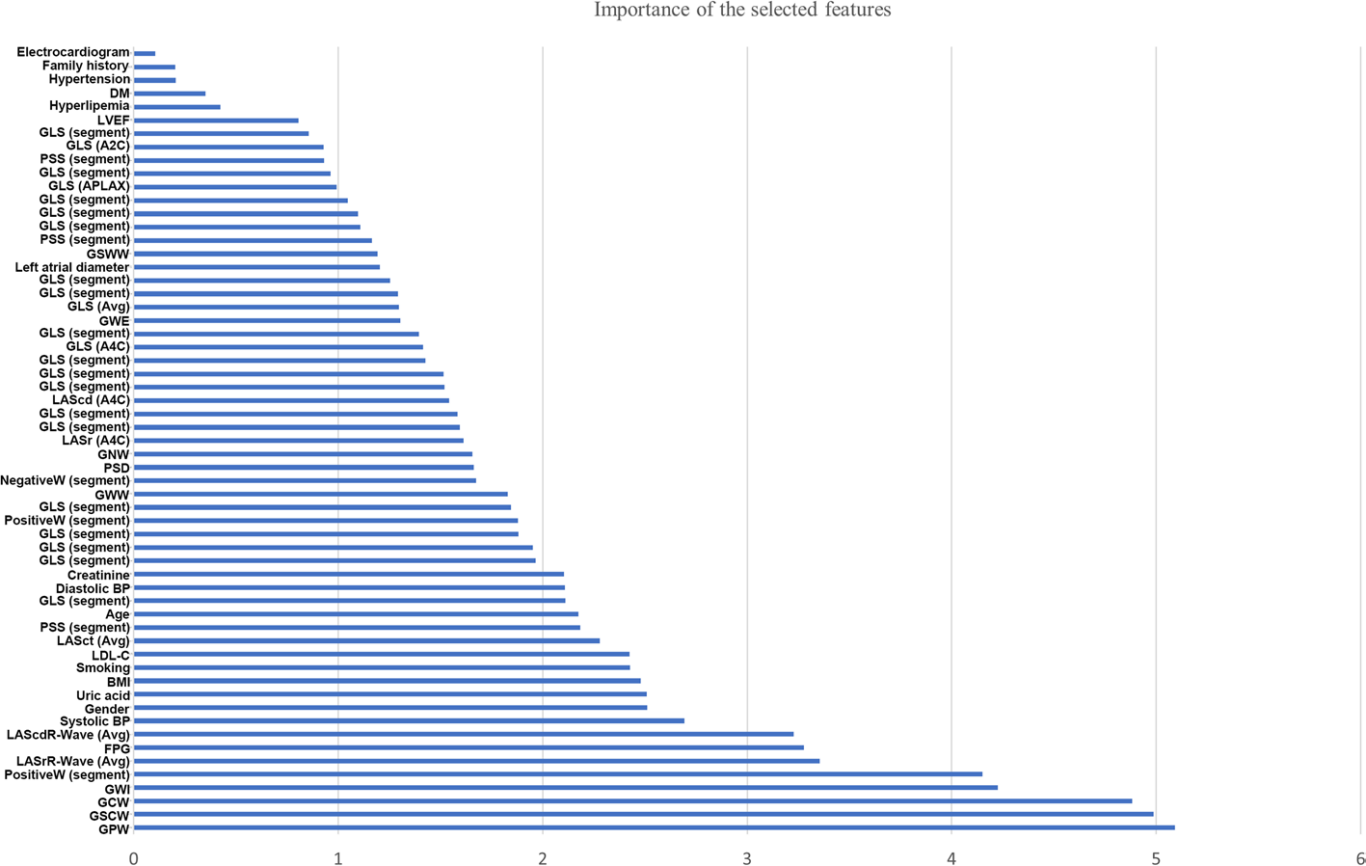
Importance of 59 selected features. *BMI* body mass index, *BP* blood pressure, *DM* diabetes mellitus, *FPG* fasting plasma glucose, *GCW* global constructive work, *GLS* global longitudinal strain, *GNW* global negative work, *GPW* global positive work, *GSCW* global systolic constructive work, *GSWW* global systolic wasted work, *GWE* global work efficiency, *GWI* global work index, *GWW* global wasted work, *LAScd* left atrial longitudinal strain during conduit phase, *LASct* left atrial longitudinal strain during contraction phase, *LASr* left atrial longitudinal strain during reservoir phase, *LDL_C* low-density lipoprotein cholesterol, *LVEF* left ventricular ejection fraction, *NegativeW* negative work, *PositiveW* positive work, *PSD* peak strain dispersion, *PSS* peak systolic strain

A stepwise strategy was adopted for the model optimization, which included feature selection, classifier selection, and training seed optimization. First, features were gradually added into the classifiers to identify a high-performance feature combination. Fifty-nine features were finally selected based on the criterion: ROC AUC should be over 80% in six decision tree classifiers, i.e., catBoost (82.9%), random forest (81.6%), gradient boosting (81.6%), extra trees (80.5%), light gradient boosting (80.4%), and Xgboost (80.4%). The distribution curve of ROC AUC for different feature combinations is shown in Fig. 3. Fifty-nine features include the clinical features: age, gender, hypertension, hyperlipemia, diabetes, family history, smoking, uric acid, creatinine, body mass index (BMI), low-density lipoprotein cholesterol (LDL_C), fasting plasma glucose (FPG), electrocardiogram, systolic blood pressure, and diastolic blood pressure, as well as the echocardiographic features: LA diameter, LVEF, GWE, GWI, GCW, GWW, GPW, GNW, GSCW, GSWW, PositiveW (2 segments), NegativeW (1 segment), PSS (3 segments), PSD, peak systolic longitudinal strain (APLAX, A4C, A2C, Avg), GLS (18 segments), LASr (A4C), LAScd (A4C), LASr (Avg), LAScd (Avg), and LASct (Avg). The evaluation of the importance of the selected features is shown in Fig. 4. Results showed that the following parameters, including GPW, GSCW, GCW, GWI, PositiveW (segment), and LASr (Avg), had incremental importance over all other parameters (including conventional echocardiographic and clinical parameters) in predicting CAD.

**Fig. 5:**
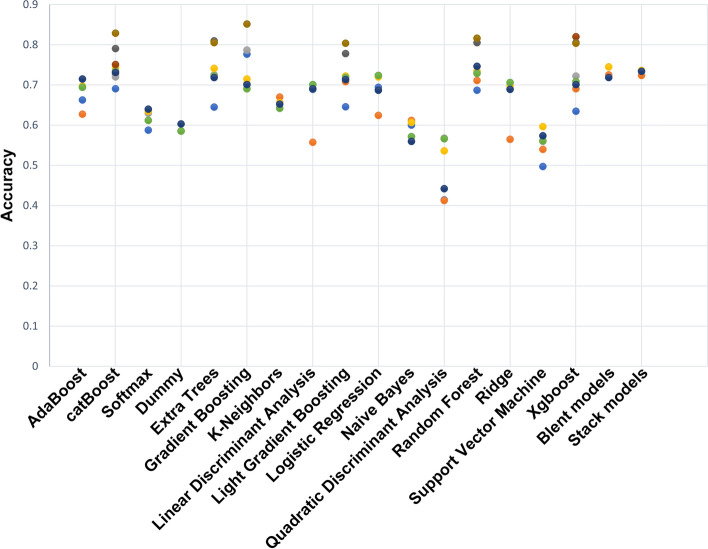
Testing accuracy of 18 classifiers using different feature combinations. The increased accuracy and superior performance (over 80% accuracy) were observed by the methods of catBoost, extra tree, gradient boosting, light gradient boosting, random forest, and Xgboost

**Fig. 6:**
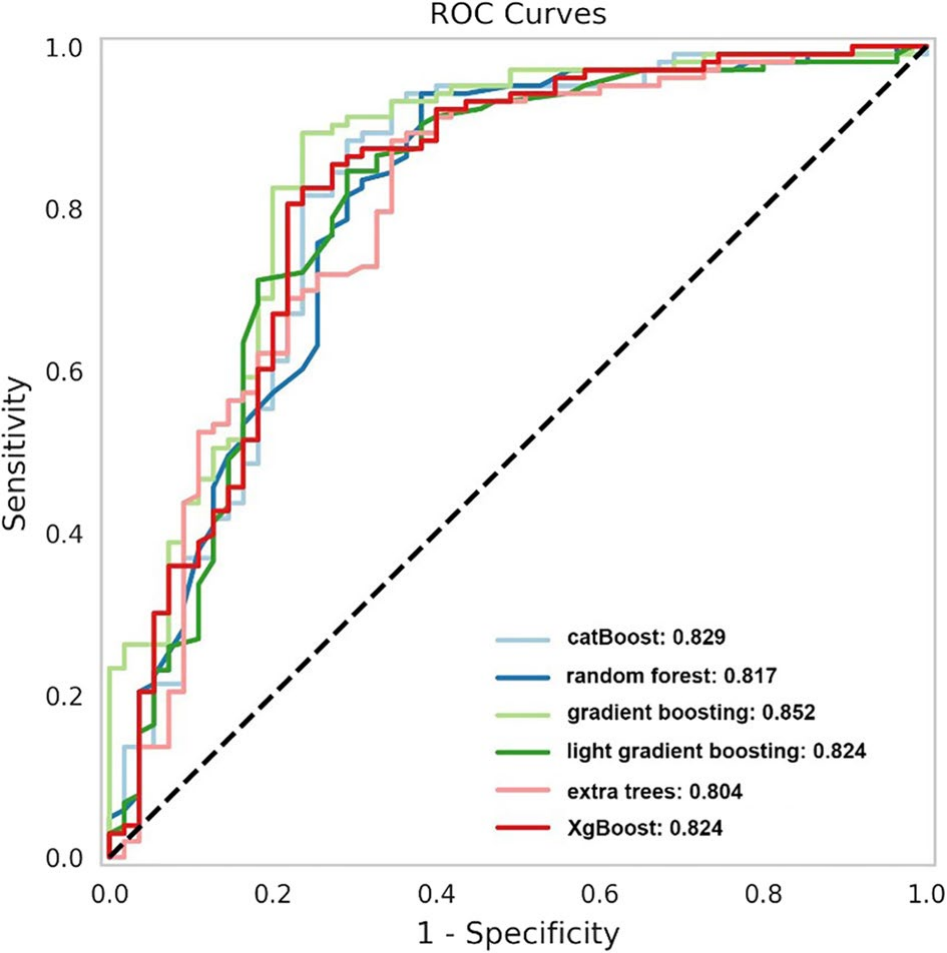
ROC curves of the proposed six classifiers with ROC AUC > 80%. Gradient boosting (light green) demonstrated the highest diagnostic efficacy among the six classifiers (ROC AUC: 0.852)

**Fig. 7:**
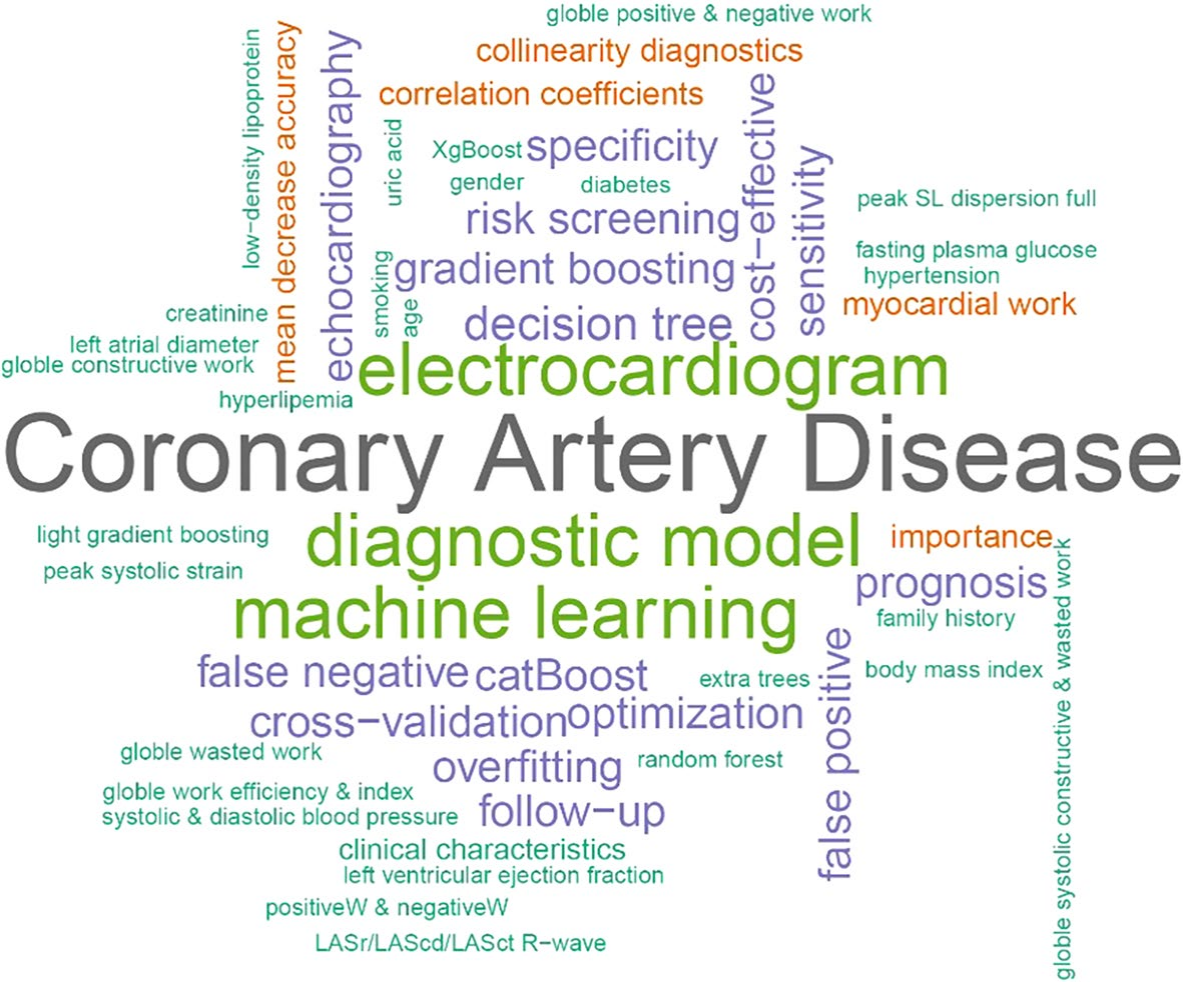
Key items of echocardiographic measurements in the machine learning model for screening CAD

In the multiple circles of cross-validation (> hundreds of times) between the training and testing data sets, we observed ROC AUC fluctuating ~ 5%, accompanied by switching training seeds. Therefore, we evaluated 635 selected training individuals and found that the accuracy of the best classifier reached 85.2% (gradient boosting), and the validation data set achieved 83.4%. The results of comparing different ML classifiers are shown in Fig. 5. In addition, the blend and stack models were established and tested for the five best classifiers. The performance of the following six ML algorithms is compared in Table 3, including catBoost, random forest, gradient boosting, extra trees, light gradient boosting, and Xgboost. Figure 6 shows the ROC curve of those six models. These results suggest that our model had superior diagnostic performance in identifying CAD patients. As shown in Fig. 7, the word cloud listed all the key points in the model construction and optimization process.

**Table 3:** ROC curve analysis of machine learning algorithms for detecting CAD

Model	Sensitivity	Specificity	ROC AUC
Gradient boosting	0.952	0.691	0.852
Catboost	0.942	0.682	0.829
Random forest	0.942	0.638	0.817
Extra trees	0.914	0.628	0.804
Light gradient boosting	0.932	0.664	0.824
Xgboost	0.932	0.664	0.824

*AUC* Area under the curve, *ROC* Receiver operator characteristics

### Clinical outcomes

Our model was very sensitive in identifying the CAD patients, with a high sensitivity (~ 95%) and a relatively low specificity (< 70%), suggesting that many non-CAD patients might be misclassified as false-positive ones. Their clinical outcomes were followed to clarify whether the false-positive patients have a high risk of CAD or a poor prognosis. The incidence of the composite endpoint stratified by our model is summarized in Table 4. The overall completeness of follow-up reached 74.9%. The composite endpoint occurred in 79 patients (13.3%) during this follow-up. The total number of composite endpoints was 110 for some reasons, such as the death of some patients, re-hospitalization at different times, or re-hospitalization for two different cardiovascular causes. In this study, 18 deaths (15 cardiac and 3 noncardiac deaths) and 92 cardiovascular hospitalizations occurred in 79 patients. The causes of death included congestive heart failure (8 patients), malignant arrhythmia (3 patients), acute myocardial infarction (2 patients), sudden cardiac death (2 patients), cancer (1 patient), and others (2 patients). Death and cardiovascular hospitalization rates were 3.0% and 10.3% at 2.5 years, respectively. Although the differences in clinical outcomes between the false-positive and other groups were not statistically significant, the trends could still be observed, based on the data that false-positive individuals (21.6%) suffered more cardiac events than that in other groups as follows: true negative (15.1%), false negative (12.5%), and true-positive (19.7%) groups (Table 4).

**Table 4:** Incidence of the composite endpoint stratified by our model

Group	CAD patients	Cases with composite endpoint	Proportion	HR (95%CI)
True-negative group	139	21	15.1	Ref
False-negative group	16	2	12.5	0.84 (0.20–3.59)
False-positive group	37	8	21.6	1.67 (0.74–3.77)
True-positive group	402	79	19.7	1.46 (0.90–2.37)

*CAD* coronary artery disease, *CI* confidence interval, *HR* hazard ratio

## Discussion

This might be the first study on the diagnostic model of CAD using novel echocardiography tools (MW and LA strain) integrated with ML models. This superior CAD detection model showed a value of ROC AUC (0.852) with a sensitivity of 0.95 and specificity of 0.69 in the test group, while the ROC AUC value was 0.834 in the validation data set. Our model was very sensitive in detecting CAD patients. We found that MW and LA strain-based ML approach has great potential to screen CAD patients.

The application of imaging techniques in clinics plays an important role in the diagnosis and prognosis of CAD, reducing the morbidity and mortality of CAD patients. Due to its feasibility and reliability, echocardiography is still the most commonly used imaging tool for diagnosing heart problems. Traditionally, the conventional echocardiographic parameter used to evaluate the presence or absence of CAD has mainly been segmental wall motion abnormalities by visualization using nude eye observation, which may miss some subtle abnormalities [46], leading to a decrease in the effectiveness of conventional echocardiography for CAD diagnosis. MW, a novel imaging tool, has many advantages compared to traditional echocardiography and other examination methods. It has been reported that the two-dimensional speckle tracking echocardiography can detect coronary stenosis, which may cause persistent myocardial dysfunction at rest [47, 48]. The strain index could be considered a marker of systolic function, which is not accurate enough (24). MW would be more accurate. The value of MW parameters in diagnosing CAD has been evaluated by previous studies [11, 40, 49], suggesting that MW is a sensitive and powerful tool for detecting advanced CAD. We used LV software to analyze LA in the past, but now we have professional LA analysis software. The present study performed LA stain analysis using a novel dedicated tracking tool. Therefore, the ML model based on the above-mentioned novel echocardiography tools may improve the efficiency of echocardiography in CAD diagnosis.

A total of 818 patients were enrolled in this study. The median sample size in the previous reports is approximately 350 [50]. There were smaller sample sizes in most studies based on echocardiography deformation imaging, because it is time-consuming and laborious to collect raw ultrasound data and perform data analysis. Most CAD patients enrolled in our study had impaired myocardial function at rest. Most MW parameters were proven to have an excellent diagnostic value for CAD (≥ 50% stenosis). In our study, GWI was a significant predictor for detecting moderate stenosis, consistent with previous reports [11, 40]. Studies on the diagnostic value of GPW and GSCW for CAD have rarely been reported. The strong features of this study include GPW, GSCW, GCW, GWI, Positive W (segment), and LASr (Avg). Our study revealed the good diagnostic value of the above-mentioned parameters for CAD. Moreover, these parameters (either conventional echocardiographic or clinical parameters) related to MW and LA strain were found to be more important in predicting CAD than all other parameters. This study has obtained valuable information through a deep learning approach. Data analysis showed no statistically significant differences in hyperlipidemia, hypertension, BMI, LDL-C, and others between the patients with and without CAD (all *P* > 0.05). The underlying differences in medication which the patients received may influence these factors. Thus, it is necessary to investigate the potential causal role of medication in the diagnosis and prognosis of CAD in further studies. Assessing the regional wall motion abnormality is very useful for detecting significant CAD patients, which has been strongly recommended [51]. In the percent study, the assessment of regional wall motion abnormality was not adopted in the final model due to its high collinearity with MW and LA strain parameters.

Coronary angiography is generally considered a gold standard for CAD diagnosis. However, coronary angiography is complex, costly, and has side effects. In contrast, the echocardiography-based methods were suitable for almost all patients, but it has been rarely reported about the clinical applications of the models that can combine novel echocardiographic methods, as mentioned above, to improve diagnostic performance. To this end, our ML model provides an approach to solving this problem. Our model demonstrated its sensitivity of 0.952 and specificity of 0.691 in the test group and its AUC of 0.852. Although the accuracy of our model was similar to that of other studies, it is notable that the inclusion criteria in our study were broader than those in the most published studies, which were closer to real-world conditions, suggesting a high sensitivity of our model, which might be used for screening the CAD patients in clinics to allow early diagnosis and treatment.

Coronary angiography mainly reflects anatomical stenoses, but it cannot reflect functional problems, while strain can partly reflect functional problems. Strain is mainly a reflection of myocardial function rather than the anatomic coronary stenosis itself. In clinical practice, many patients had myocardial ischemia without obstructive CAD [52, 53]. Thus, doctors often misdiagnose noncardiac chest pain based on normal or nearly normal coronary arteriograms. Actually, anginal symptoms might be caused by several mechanisms, such as coronary spasms and/or microvascular dysfunction [54]. This may be the reason for the relatively high false-positive rate in our study when the new model test is positive, but the gold standard is negative. Our model promised 0.852 ROC AUC with a sensitivity of 0.95 and specificity of 0.69 in the test group, suggesting that it is a very sensitive tool to detect CAD, but non-CAD patients may be misclassified (resulting in a high false-positive rate) by its low specificity. A total of 74.9% of patients in our study completed the follow-up. Even though our median follow-up time is 2.5 years, some trends were still determined. Combined with the prognosis, we found that the false-positive patients who were identified by the model were more susceptible to suffering cardiac events than the true-negative patients. Furthermore, compared to the true-positive patients, the false-positive patients experienced more cardiac events, which may be explained by the fact that many of the true-positive patients had already received a percutaneous coronary intervention. This study confirmed the potential predictive value of the new model on cardiac events. In addition, our model's potential clinical value is to evaluate patients with normal or nearly normal coronary arteriograms.

ML algorithms have been widely used for analyzing medical images [31, 55–57]. Clinical application of our model built by ML algorithms can improve the accuracy of diagnosis, reduce the input of experts, save cost and time, and ultimately provide high-quality patient services. This model also helps rule out patients without coronary heart disease and avoid unnecessary coronary angiography. Compared with MRI, SPECT, and other time-consuming examination methods, the clinical application of this model could provide diagnostic results in a shorter time. The potential clinical applications of the echocardiography-based ML model have been reported in recent years, but safer and more effective methods for diagnosing and assessing the CAD prognosis are still expected. Indeed, our method may provide a more efficient and non-invasive way for early screening and diagnosis of CAD, which may remarkably improve the diagnostic modality of non-invasive imaging methods. This technique may also support the pre-test CAD probability assessment in outpatient clinics or CAD screening in physical examination centers.

### Limitations

This study had several limitations. It was a single-center study due to its data collected from the same medical system. In this study, we enrolled patients who underwent coronary angiography with typical myocardial ischemia-related symptoms or positive results of examinations. In addition, a single echocardiogram vendor and post-processing algorithm were applied. All of those may increase the instability of the model, resulting in low generalization of the results. Therefore, further studies with multi-center data will be considered, in which the method of an adaptive learning process could be included, enabling the model to update when inputting new samples automatically. The semi-automatic speckle tracking analysis was another limitation of our study. Because of this, the different physicians' subjective opinions may also influence the final prediction. The difficulty to recognize the epicardial or endocardial border by EchoPac (in case of processing low-quality images) was also a critical issue in our model, which could bring certain biases to the results. To address this issue, it is necessary to develop an automatic image quality control and tracing technique to analyze echocardiogram data. To effectively reduce subjective errors, some efforts should be made to reduce user intervention in image feature extraction and classification analysis. Because our overall follow-up time was limited, we have only discovered a few trends thus far. We will continue to follow up with these patients. A risk stratification assessment model will be established based on the prognosis of those patients.

## Conclusions

Our study demonstrated the following benefits of our model in CAD diagnosis: (1) good diagnostic performance in screening CAD patients, confirmed in the validation group; and (2) the predictive function of our model only requires the non-invasive echocardiographic and some commonly used clinical features. In summary, our novel model could provide a more efficient and non-invasive method for screening and diagnosing CAD in clinics.

## Data Availability

The data sets used and/or analyzed during the current study are available from the corresponding author upon reasonable request.

## Abbreviations

AI: Artificial intelligence
AUC: Area under the curve
BMI: Body mass index
BP: Blood pressure
CAD: Coronary artery disease
CI: Confidence interval
ConsW: Constructive work
DM: Diabetes mellitus
FPG: Fasting plasma glucose
GCW: Global constructive work
GLS: Global longitudinal strain
GNW: Global negative work
GPW: Global positive work
GSCW: Global systolic constructive work
GSWW: Global systolic wasted work
GWE: Global work efficiency
GWI: Global work index
GWW: Global wasted work
HR: Hazard ratio
LA: Left atrial
LAScd: Left atrial longitudinal strain during conduit phase
LASct: Left atrial longitudinal strain during contraction phase
LASr: Left atrial longitudinal strain during reservoir phase
LDL_C: Low-density lipoprotein cholesterol
LV: Left ventricular
LVEF: Left ventricular ejection fraction
MDA: Mean decrease accuracy
ML: Machine learning
MW: Myocardial work
NegativeW: Negative work
PositiveW: Positive work
PSD: Peak strain dispersion
PSS: Peak systolic strain
ROC: Receiver operating characteristic
SysConsW: Systolic constructive work
SysWastedW: Systolic wasted work
TDI: Tissue doppler imaging
WastedW: Wasted work
